# X-linked myotubular myopathy in a neonate: a case report and literature review

**DOI:** 10.3389/fped.2026.1735095

**Published:** 2026-03-30

**Authors:** Lin Chen, You-Qun Zou

**Affiliations:** Department of Neonatology, Guangdong Provincial People's Hospital, Guangdong Academy of Medical Sciences, Southern Medical University, Guangzhou, China

**Keywords:** MTM1 mutation, neonatal hypotonia, respiratory failure, whole-exome sequencing, x-linked centronuclear myopathy

## Abstract

**Background:**

X-linked centronuclear myopathy (XLCNM) is a rare congenital neuromuscular disorder caused by pathogenic variants in *MTM1*, typically presenting with severe neonatal hypotonia, respiratory failure, and poor survival. Early diagnosis is essential for prognosis and genetic counseling, though clinical recognition is often challenging.

**Case presentation:**

We report a male infant, born at 38 + 1 weeks via cesarean section, who presented immediately after birth with apnea, cyanosis, hypotonia, and poor responsiveness. Despite resuscitation and intensive care for neonatal asphyxia and sepsis, he remained ventilator-dependent with persistent hypotonia and feeding difficulties. Laboratory evaluation showed anemia of chronic disease, hypoproteinemia, vitamin D insufficiency, and mild hepatic dysfunction. Imaging revealed laryngomalacia and a patent foramen ovale. Given the family history of neonatal death in a male sibling, whole-exome sequencing (WES) was performed and identified a hemizygous nonsense mutation in *MTM1* (NM_000252.2): c.373C > T (p.Gln125Ter), confirming XLCNM. Maternal heterozygosity was verified by Sanger sequencing, consistent with X-linked recessive inheritance. In view of the severe clinical course and poor prognosis, the family declined further treatment. The patient was discharged for palliative care and died shortly after from respiratory failure.

**Conclusion:**

This case reveals the importance of early recognition and genetic diagnosis of XLCNM, particularly in neonates with unexplained hypotonia and a suggestive family history. Through our literature review, we emphasize the need for heightened clinical awareness, summarize current therapeutic and research advances, and discusses supportive strategies that may optimize survival and quality of life in affected infants.

## Introduction

Centronuclear myopathy (CNM) is a rare congenital myopathy with clinical and genetic heterogeneity, named for its characteristic histopathological finding of numerous muscle fibers containing centrally located nuclei (1). CNM is characterized histologically by centrally placed nuclei within myofibers, often with accumulation of mitochondria in the central region of small fibers. The most common causative genes include *DNM2, BIN1, MTM1*, and *RYR1*, with others such as *TTN, SPEG, CACNA1S*, and *ZAK* also implicated (2). Based on inheritance patterns, CNM can be classified as X-linked recessive, autosomal dominant, autosomal recessive, or sporadic (3). Sporadic CNM is a rare and heterogeneous neuromuscular disorder characterized by centrally located nuclei in muscle fibers. A sporadic presentation is often caused by a *de novo* gene variant, most commonly in DNM2, although late-onset cases without a clear family history may also occur (4, 5). X-linked CNM (XLCNM, myotubular myopathy) is the most severe phenotype and results from pathogenic variants in the *MTM1* gene (6). This form accounts for approximately 57% of CNM cases (7). *MTM1* gene encodes myotubularin, a phosphatase essential for normal skeletal muscle development and function. Loss-of-function mutations result in profound muscle weakness and multisystem involvement. XLCNM typically affects male infants, while female carriers are usually asymptomatic or have milder symptoms. In most familial cases, the mother is an asymptomatic carrier. When a positive family history is present, prenatal diagnosis of XLCNM is possible. The incidence of XLCNM is estimated at approximately 1 in 50,000 live male births, representing the largest proportion of CNM cases, with a generally poor prognosis (8, 9).

Clinical severity can range from mild weakness to profound neonatal hypotonia with respiratory failure. In its most severe form, symptoms present at birth and include generalized muscle weakness, feeding difficulties, impaired respiratory effort, and delayed motor development. Complications such as skeletal deformities, recurrent respiratory infections, and ventilator dependence are common. Most severely affected patients die within the first month of life. Carrier females may present with limb-girdle or facial weakness (10).

Currently, there is no curative therapy for XLCNM. Management is supportive and multidisciplinary, focusing on optimizing respiratory function, providing nutritional assistance, preventing complications, and offering genetic counseling (11, 12). Experimental approaches, including gene replacement therapy, are under investigation and hold promise for altering the disease course.

In the present report, we describe a neonate with a history of birth asphyxia who developed generalized hypotonia, muscle weakness, respiratory failure, and feeding difficulties immediately after birth. Despite intensive supportive care, there was no improvement. Whole-exome sequencing (WES) confirmed an *MTM1* pathogenic variant consistent with XLCNM. Despite intensive supportive care, there was no improvement, and the infant died at 62 days of life following withdrawal of treatment at parental request. Moreover, we reviewed 21 published reports from the past decade focusing on neonatal- and childhood-onset XLCNM. Most infants presented with polyhydramnios, reduced fetal movements, profound hypotonia, and ventilatory dependence, with survival rarely beyond the first year despite intensive care. Supportive measures such as tracheostomy, gastrostomy, and multidisciplinary management prolonged survival in a minority. This case and literature review reveal the importance of early recognition, genetic testing, and counseling to guide diagnosis, reproductive planning, and supportive care.

## Case presentation

A male infant, G3P3 (gravida 3 para 3), was delivered at 38 + 1 weeks of gestation at local hospital via elective cesarean section for scarred uterus. Amniotic fluid was clear, with no umbilical cord entanglement. Apgar scores were 2, 7, and 9 at 1, 5, and 10 min, respectively. Birth weight was 2,950 g (25th percentile for 38 + 1 weeks of gestation). Birth length was 50 cm (SD 0 to +1). Head circumference was 33.5 cm (SD −1–0). Immediately after birth, the infant exhibited apnea, cyanosis, hypotonia, poor responsiveness, and bradycardia (∼40 bpm). Resuscitation included airway clearance, tracheal intubation, positive pressure ventilation, and chest compressions. He was admitted to the local NICU with diagnoses of severe neonatal asphyxia, neonatal shock, and sepsis. Initial management comprised invasive mechanical ventilation, non-invasive respiratory support, volume expansion, empirical antibiotics (ceftazidime, subsequently escalated to meropenem), intravenous immunoglobulin, and supportive therapy. Despite partial improvement, he remained ventilator-dependent, with marked intercostal and sub-sternal retractions and minimal oral intake. He was transferred to our center for further evaluation. The transport was uneventful.

On admission to our center, the infant's vital signs were temperature 37.1 °C, heart rate 167 bpm, blood pressure 57/36 mmHg, and weight 2,940 g (25th percentile for age). Physical examination revealed coarse breath sounds with moist rales, a grade II cardiac murmur, hypotonia, absent primitive reflexes, and bilateral undescended testes. No dolichocephaly, high-arched palate, ptosis, or facial dysmorphism was observed. Subsequent laboratory and imaging evaluations revealed persistent anemia (hemoglobin 95 g/L; newborn: 140–240 g/L) with low red blood cell counts and reactive thrombocytosis (platelets up to 522 × 10⁹/L), consistent with anemia of chronic disease rather than iron deficiency, as iron studies were normal. White blood cells and inflammatory markers initially elevated and gradually normalized, indicating resolution of infection. Endocrine and nutritional assessments demonstrated vitamin D insufficiency [25(OH)D 24.4 ng/mL], transient thyroid function abnormalities (elevated free T4 with normal TSH), and hypoproteinemia (albumin 30.1 g/L). Biochemical testing showed mild hepatic dysfunction, including elevated ALT/AST, hypoalbuminemia (albumin 30.1 g/L), and hyperbilirubinemia (total bilirubin 181.2 μmol/L; direct bilirubin 21.9 μmol/L), with low albumin), but renal function and electrolytes were largely preserved. Imaging identified a patent foramen ovale (2.5 mm, left-to-right shunt) on echocardiography. Abdominal and inguinal ultrasonography confirmed bilateral undescended testes (cryptorchidism). Bronchoscopy revealed tongue base collapse with mild laryngomalacia, consistent with ongoing upper airway obstruction, while chest radiographs demonstrated bilateral pulmonary infiltrates. Muscle enzymes (CK, CK-MB) were not significantly elevated, and no major metabolic derangements were noted. Diagnostic evaluation included detailed clinical examination, laboratory testing, imaging studies, bronchoscopy, and genetic testing using WES with Sanger confirmation. The presence of severe neonatal asphyxia and sepsis initially complicated the diagnosis, as hypotonia and respiratory failure could be attributed to hypoxic-ischemic injury. Normal muscle enzyme levels further delayed suspicion of a primary neuromuscular disorder. Differential diagnoses included hypoxic-ischemic encephalopathy, spinal muscular atrophy, congenital myopathies, metabolic disorders, and chromosomal abnormalities. Persistent ventilator dependence, profound hypotonia, absent primitive reflexes, cryptorchidism, and a family history of neonatal male death raised suspicion of an inherited neuromuscular disorder, prompting genetic testing.

The patient was managed with invasive mechanical ventilation (SIMV mode, PIP 20 cmH_2_O, PEEP 6 cmH_2_O, respiratory rate 40 breaths/min, FiO_2_ 28%). Airway management included regular endotracheal suctioning, humidified support, chest physiotherapy, and postural drainage. Empiric antibiotics (ceftazidime, later meropenem, then piperacillin–tazobactam) were administered. Blood transfusion corrected anemia. Nutritional support was provided via nasogastric feeding with a caloric intake of approximately 100–120 kcal/kg/day, along with vitamin D supplementation. After extubation, the patient was transitioned to non-invasive ventilatory support; however, persistent tachypnea, intercostal retractions, and supine positional dyspnea necessitated continued respiratory assistance. Bronchoscopy demonstrated tongue base collapse and mild laryngomalacia. Positioning therapy and intensified airway clearance were implemented. Despite these measures, the patient remained ventilator-dependent with no improvement in muscle strength or tone.

**Figure 1:**
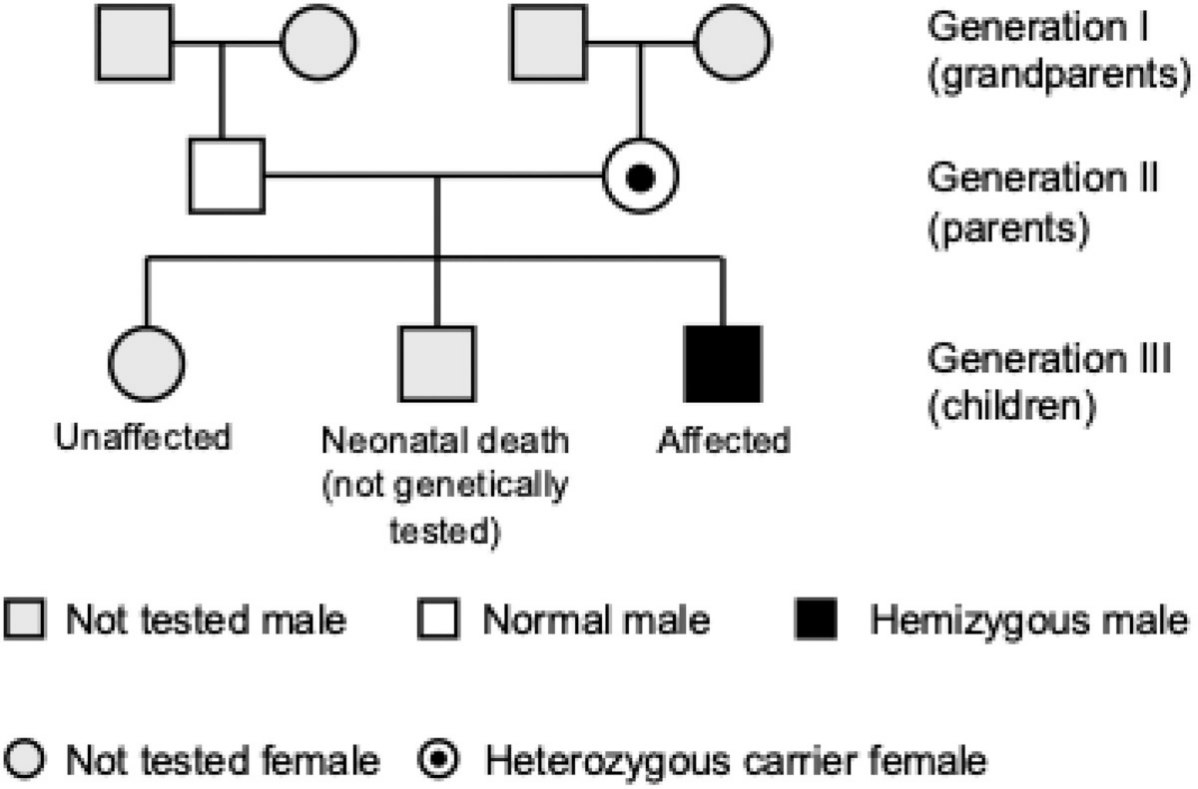
Pedigree of the family with XLCNM. The proband (Generation III) is a hemizygous affected male carrying the MTM1 variant (c.373C > T, p.Gln125Ter). The mother is a heterozygous carrier. An older male sibling died in the neonatal period due to respiratory failure and was not genetically tested. Squares indicate males and circles indicate females. Filled symbols represent affected individuals. A circle with a central dot indicates a heterozygous carrier. Open symbols indicate unaffected or not genetically tested individuals.

**Figure 2:**
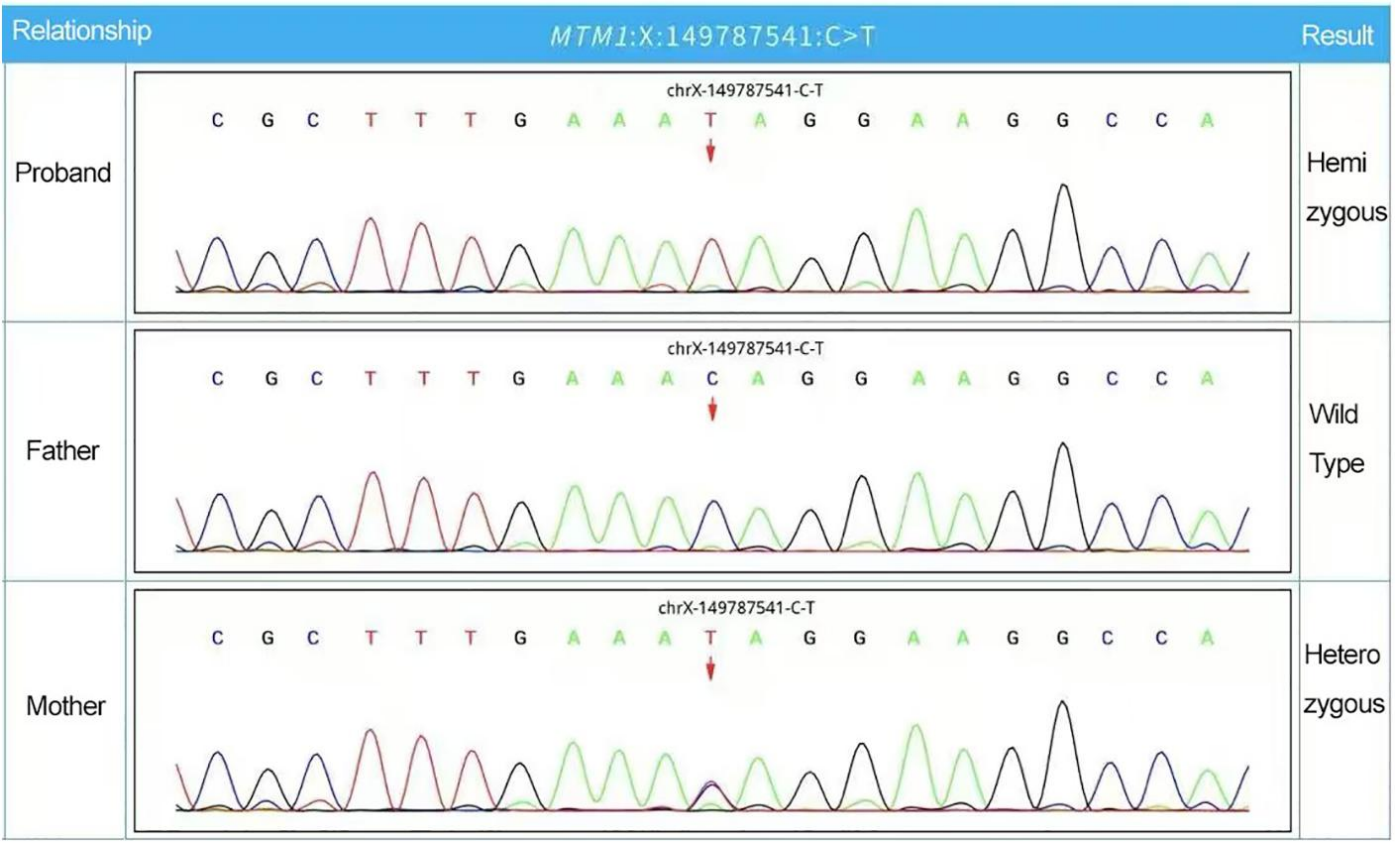
Sanger sequencing chromatograms showing the MTM1 variant (c.373C **>** T, p.Gln125Ter). The proband demonstrates a hemizygous C > T substitution. The mother shows heterozygosity, while the father has a normal sequence.

Given the family history of neonatal death in a male sibling, further genetic evaluation was discussed repeatedly with the parents. After informed consent was obtained, rapid WES was performed at 37 days of age using peripheral blood samples from the patient and his parents The turnaround time from sample submission to final report was 8 days. The analysis identified a hemizygous nonsense mutation in *MTM1* (NM_000252.2): c.373C > T (p.Gln125Ter) at chrX:149787541. This variant introduces a premature termination codon and is predicted to result in loss of function, a well-established disease mechanism in MTM1-related XLCNM (12). The variant was not found in ClinVar or population databases, including gnomAD, at the time of analysis and is therefore considered novel. The variant was classified as pathogenic according to ACMG/AMP guidelines based on PVS1 (null variant in a gene where loss-of-function is a known disease mechanism) and PM2 (absence from population databases) (13, 14). A three-generation pedigree illustrating the inheritance pattern is shown in Figure 1. Sanger sequencing verified maternal heterozygosity, consistent with X-linked recessive inheritance (Figure 2).

Despite active treatment, the patient remained ventilator-dependent with no improvement in muscle strength or tone. After confirmation of the genetic diagnosis, the medical team conducted detailed discussions with the parents regarding the prognosis, including the need for long-term ventilatory support and the reported mortality risk within the first year of life. We also explained that the disease is not typically progressive and that cognitive development is often preserved in the absence of significant hypoxic brain injury. After comprehensive counseling and consideration of family circumstances, the parents elected to withdraw life-sustaining treatment. The decision was made by the parents in accordance with institutional regulations, and written informed refusal of continued ventilatory support was obtained. Mechanical ventilation was withdrawn. The patient was transitioned to low-flow nasal oxygen therapy and continued to receive supportive care, including airway suctioning, nutritional support, and vitamin supplementation. After 8 days, patient was discharged home for comfort-oriented care. The infant died (63-day old) from respiratory failure the following day.

## Discussion

XLCNM is among the most severe congenital myopathies, typically presenting in the neonatal period with respiratory failure, hypotonia, and feeding difficulties (8, 9). Our patient exhibited the classic phenotype, including profound hypotonia, absent primitive reflexes, ventilator dependence, and family history of male sibling death, which collectively prompted suspicion of an inherited neuromuscular disorder. WES confirmed a hemizygous nonsense mutation in *MTM1* (NM_000252.2): c.373C > T (p.Gln125Ter), a well-recognized pathogenic variant leading to premature termination of the protein. Truncating variants in MTM1 are typically associated with the classic severe neonatal phenotype, characterized by profound hypotonia, respiratory failure at birth, and high mortality in infancy. The identification of maternal heterozygosity further supported X-linked recessive inheritance.

To better understand the clinical spectrum and prognosis of XLCNM, we reviewed 21 case reports published in the past ten years with sufficient clinical detail regarding presentation, management, and outcomes, focusing on neonatal- and childhood-onset cases (Table 1) (15–27). Polyhydramnios emerged as a consistent antenatal finding in many cases, often accompanied by reduced fetal movements or structural changes such as cervical skin thickening, pleural effusions, or scaphocephaly. Most affected infants were delivered at term, though several reports described preterm deliveries (ranging from 29 to 36 weeks). It has been reported that about 80% of affected males with the classic form of XLCNM present with polyhydramnios (28). These findings underscore the importance of prenatal surveillance when polyhydramnios is accompanied by sonographic evidence of neuromuscular compromise.

**Table 1:** Reported cases of XLCNM with neonatal, infantile, and childhood onset.

Case	Sex	Diagnosis	Antenatal and gestation	Initial clinical features	Treatment	Outcomes
1	M	MTM1 mutation (c.373C > T, p.Gln125Ter)	Term (38 + 1 weeks)	Severe hypotonia; absent primitive reflexes; feeding difficulty; failed extubation (tachypnea, retractions, distress in supine position), paradoxical breathing; recurrent pneumothorax. tongue base collapse; mild laryngomalacia; grade II murmur. Other findings: Hepatomegaly, bilateral cryptorchidism.	Mechanical ventilation; antibiotics (ceftazidime, meropenem, later piperacillin, tazobactam); intravenous immunoglobulin; airway clearance; postural drainage, neurotrophic therapy; vitamin D supplementation.	Demised at 62 days of life.
2 (15)	M	Muscle biopsy	Polyhydramnios; preterm (34 + 4 weeks)	Generalized hypotonia at birth; rest unknow.	Mechanical ventilation from birth; palliative tracheostomy at 6 weeks of life.	Demised at 9 weeks of life.
3 (15)	M	MTM1 mutation (c.343-1G > A, intron 5)	Polyhydramnios; cervical skin thickening; initial pleural effusions (resolved by 23 weeks); term (37 + 1 weeks).	Generalized hypotonia at birth; spontaneous antigravity movement noted; absent gag and deep tendon reflexes; high arched palate, long fingers and toes, bilateral undescended testes; chest x-ray finding of thin ribs.	Extubated on day 4 of life to non-invasive ventilation	Demised at 1 year 9 months old.
					Discharged at 6 weeks of age and remained on non-invasive ventilation and nasogastric tube feeding.	
4 (16)	M	MTM1 mutation (c.868-2A > C, intron 9)	Polyhydramnios; late preterm (36 + 2 weeks).	Generalized hypotonia at birth; no response to stimulation; bradycardia (heart rate < 60 bpm).	Immediate resuscitation at birth; invasive mechanical ventilation (prolonged, weaning unsuccessful); anti-infective therapy (antibiotics for pneumonia); correction of metabolic derangements (acidosis, hypocalcemia).	Demised at 15 days of life.
5 (17)	M	MTM1 mutation (c.1261A > G)	Term (39 weeks)	Difficulty in breathing and swallowing; severe generalized hypotonia; slender fingers and toes; preserved primitive reflexes.	Mechanical ventilation, supportive therapy (antibiotics, IV nutrition, tube feeding); multiple failed extubation attempts; persistent swallowing dysfunction despite rehabilitation.	Demised at 21 days of life.
6 (17)	M	MTM1 mutation (c.342delT)	Polyhydramnios; term (38 + 4 weeks)	Difficulty in breathing and swallowing; severe generalized hypotonia.	Continued ventilation, tube feeding, infection prevention, rehabilitation attempts. Persistent swallowing dysfunction.	Demised at 48 days of life.
7 (18)	M	MTM1 mutation (c.1261-5T > G, exon 12)	Polyhydramnios; term (39 + 6 weeks)	Generalized hypotonia at birth; respiratory distress with pulmonary hypertension.	Mechanical ventilation at birth; later tracheostomy; gastrostomy.	Demised at 13 months old.
8 (19)	M	MTM1 mutation (c.1262G > A, p.R421Q)	Not reported	Hyperbilirubinemia; pulmonary atelectasis.	Neonatal jaundice: Phototherapy (no exchange transfusion required); cholestasis management (7.5–10 months): ursodeoxycholic acid (UDCA) 17 mg/kg/day, fat-soluble vitamins, and antihistamines (for pruritus); Nighttime non-invasive ventilation for atelectasis.	Demised at 12 months due to cardiopulmonary failure.
9 (19)	M	MTM1 mutation (c.1420C > T, p.R474*)	Not reported	Respiratory distress; cholestasis episode at 8 months; febrile lower respiratory tract infection; pseudomonas respiratory infection; longstanding intense pruritus, hepatic-related.	Long-term mechanical ventilation; gastrostomy; cholestasis/Pruritus: UDCA 18 mg/kg/day, cholestyramine (initially 800 mg q12 h, later maintained at 2.4 g/day), and fat-soluble vitamins; antimicrobials: IV gentamicin, ciprofloxacin, and colistin.	At last follow-up: stable.
10 (19)	M	MTM1 [79 kb deletion (chrX:149761067-149840078, exons 3–14)]	Not reported	Respiratory distress; at 14 months: acute scleral icterus, pruritus, choluria, hypocholic stools after infection.	Mechanical ventilation; gastrostomy; UDCA 10–15 mg/kg/day; cholestyramine for pruritus (dose adjusted/reduced due to metabolic acidosis); respiratory infections (oral amoxicillin).	Recurrent episodes of cholestasis and pruritus; remained ventilator- and gastrostomy-dependent.
11 (19)	M	MTM1 mutation (c.1644 + 1G > A)	Not reported	Respiratory distress; at 16 months: jaundice, pruritus, choluria (no acholic stools, no hepatomegaly), onset after pneumococcal and MMR vaccinations.	Mechanical ventilation; gastrostomy; UDCA 15 mg/kg/day; hydroxyzine 1 mg/kg/day (for pruritus).	Stable with ongoing ventilator and gastrostomy dependence.
12 (19)	M	MTM1 mutation (c.1088_1089del p.K363Sfs*14)	Not reported	Respiratory distress; at 5 months: jaundice; progressive severe intrahepatic cholestasis.	Mechanical ventilation; supportive care for chronic liver disease.	Liver failure due to fibrosis.
13 (20)		Muscle biopsy	Not reported	Poor eye opening, myopathic facies; pooling of pharyngeal secretions; mild contractures (elbows, knees); generalized hypotonia (axial > appendicular); decreased muscle bulk; anti-gravity movement present in all extremities; absent deep tendon reflexes; feeding difficulty and aspiration.	Continuous positive airway pressure (CPAP) for upper airway hypotonia; BiPAP by 2 months (respiratory failure); Gastrojejunostomy tube; Emergency deterioration: developed hypoxemia, intracranial hemorrhage, hydrocephalus, profound coagulopathy (vitamin K deficiency-related cholestasis), required vasopressors and transfusions.	Poor prognosis declared after massive intracranial hemorrhage and metabolic derangements; comfort measures pursued, life-sustaining therapies withdrawn; feeding difficulty and aspiration.
14 (21)	M	MTM1 mutation (c.1468-577A > G)	Polyhydramnios	Severe central and peripheral hypotonia with scaphocephaly, myopathic facies, congenital ptosis, marked ophthalmoplegia, severe facial weakness (unable to close eyelids in sleep), cryptorchidism, small chest with thin ribs, mild hip dysplasia, and absent respiratory effort.	Tracheostomy at 4 months; percutaneous endoscopic gastrostomy (PEG) tube at 10 weeks; ventilatory support; surgical fixation for severe kyphoscoliosis at 12 years; myringotomy tubes.	Alive.
15 (22)	M	MTM1 mutation (c.1189dupT p.Tyr397fs); and DMD (Xp21.1 deletion, exons 46–52)	Preterm (31weeks)	Floppy, no respiratory effort but good heart rate; persistent poor respiratory effort despite minimal ventilator support, failed extubation attempts; hypotonia with paucity of limb movements; myopathic facies and facial weakness; poor gag and suck reflexes; absent/depressed deep tendon reflexes; bilateral undescended testes.	Immediate intubation and surfactant; prolonged mechanical ventilation; nutritional support: low-LCT, high-MCT formula for chylothorax.	Demised at 2.5 months.
16 (23)	M	Muscle biopsy + genetic analysis	Not reported	Diagnosed with XLCNM in early childhood; recurrent pneumothorax; at 9 years 4 months: presented with vomiting, tachycardia, pallor, and hypovolemic shock due to massive intra-abdominal hemorrhage from the liver.	Ventilator dependence; gastrostomy; emergency left lateral segmentectomy (initial control of bleeding); ongoing bleeding: gauze packing + right hepatic artery ligation; emergency living-donor liver transplantation using graft from father.	At 10-month follow-up: patient alive, well, and without recurrence of peliosis hepatis.
17 (23)	M	Muscle biopsy	Not reported	Diagnosed with XLCNM in infancy; at 1 year 7 months: worsening respiratory status, tachycardia, anemia.	Emergency transcatheter arterial embolization (TAE); living-donor liver transplantation using graft from father; immunosuppression: tacrolimus, low-dose steroids, mycophenolate mofetil.	At 9-month follow-up: stable, satisfactory condition in outpatient care.
18 (24)	M	MTM1 mutation (c.1420C > T p.Arg474*)	Term (37 weeks)	Severe neonatal hypotonia and extremely weak spontaneous respiration; difficulty in breathing and swallowing.	Long-term ventilator dependence, NG/G-tube feeding, vitamin K, leukotriene receptor antagonist, expectorant; emergency resuscitation after tracheostomy accident, blood transfusions for hemorrhage; espite interventions, developed hypovolemic shock and progressive liver failure.	Demised at 4 years old.
19 (25)	M	MTM1 mutation (c.527A > G p.Gln176Arg)	Not reported	Severe neonatal asphyxia; high-arched palate; generalized muscle weakness; no head control, but could sit independently at 5 years.	Mechanical ventilation since birth; tracheostomy at 7 months; gastrostomy at 4 years.	Still alive at time of report (5 years old).
20 (25)	M	MTM1 mutation (c.688T > C p.Trp230Arg)	Not reported	Severe neonatal asphyxia, respiratory failure soon after birth; generalized muscle weakness; no head control.	Extubated at 7 days, but still had generalized muscle weakness.	Still alive at time of report (5 months old).
21 (26)	M	79 kb deletion in MTM1 (exons 3–14)	Preterm (29 weeks)	Severe floppiness and poor respiration at birth; severe generalized hypotonia; pectus excavatum, tachypnea, paradoxical breathing; absent deep tendon reflexes and gag reflex; no tongue fasciculations.	Immediate intubation at birth; mechanical ventilation; mechanical insufflation-exsufflation (MI-E, +40/−40 cmH₂O); respiratory physiotherapy/rehabilitation.	At 2 years 6 months (latest follow-up): still bedridden without head control; can turn body/face side to side, raise arms.
22 (27)	M	Muscle biopsy + genetic analysis	Polyhydramnios; term (37 weeks)	Severe birth asphyxia accompanied by hypotonia and dyspnea; at 10 days old: a respiratory complication of chylothorax.	Mechanical ventilation at birth; thoracic drainage at 10 days old; Tracheostomy at 5 months.	Discharged at 17 months old.

Early diagnosis of XLCNM is crucial, as neonatal hypotonia and respiratory insufficiency can mimic more common etiologies such as hypoxic-ischemic encephalopathy, sepsis, or metabolic disease (29). In settings where genetic testing is not readily available, recognition of associated clinical features may help raise suspicion. Beyond profound hypotonia, commonly reported findings include facial muscle involvement (high-arched palate, congenital ptosis, ophthalmoplegia, and myopathic facial appearance), cryptorchidism, and occasionally neonatal jaundice with transient hepatic dysfunction (19, 28). In our patient, bilateral undescended testes were identified on physical examination and confirmed by ultrasonography, supporting the clinical suspicion of an inherited neuromuscular disorder. The coexistence of severe hypotonia, respiratory failure, cryptorchidism, and characteristic craniofacial features should prompt consideration of XLCNM, even in the absence of a known family history. As shown in Table 1, severe generalized hypotonia was the hallmark presentation, often accompanied by absent or depressed primitive reflexes, poor spontaneous respiration, and feeding difficulties. Respiratory failure at birth was common, with many infants requiring immediate resuscitation and mechanical ventilation. Additional features included high-arched palate, long fingers and toes, cryptorchidism, hepatomegaly, congenital ptosis, and ophthalmoplegia, reflecting the multisystem involvement of XLCNM. Interestingly, a subset of patients demonstrated mild preserved antigravity movements or reflexes, which correlated with slightly longer survival. In this case, the persistence of ventilator dependence without neurological recovery, coupled with a family history of neonatal death, indicated the importance of considering genetic myopathies early in the differential diagnosis. Advances in next-generation sequencing have allowed for more rapid recognition.

Respiratory support was the cornerstone of management. Most patients required invasive mechanical ventilation from birth, with several undergoing tracheostomy for long-term support. Non-invasive ventilation was successful in a minority of cases and often allowed brief periods of survival beyond infancy. Supportive therapies included antibiotics for recurrent respiratory infections, airway clearance techniques, nutritional support via nasogastric or gastrostomy tubes, and in some reports, immunoglobulin or vitamin supplementation. In long-term survivors, surgical interventions such as gastrostomy placement, tracheostomy, and spinal fixation are often required.

Our patient's course reflects these challenges, particularly the burden of prolonged ventilatory dependence and feeding difficulties. Despite advances in neonatal intensive care, the overall prognosis remains poor. Most infants do not survive beyond the first year of life, while a minority may reach late infancy or early childhood with persistent ventilator and feeding tube dependence. Rare long-term survivors were reported, some developing severe complications such as cholestasis, liver failure, or peliosis hepatis requiring transplantation. These cases reveal both the systemic burden of XLCNM and the potential role of multidisciplinary care in prolonging survival. Given these outcomes, genetic counseling is crucial for families with a history of XLCNM. Preimplantation genetic testing (PGT), performed in conjunction with *in vitro* fertilization (IVF), offers a valuable reproductive option for families carrying known MTM1 variants. Through embryo biopsy and genetic analysis at the blastocyst stage, unaffected embryos can be selected for transfer, thereby preventing recurrence. Different approaches, including PGT-A for aneuploidy, PGT-M for monogenic disorders such as MTM1 mutations, and PGT-SR for structural rearrangements, allow tailored screening depending on the clinical context (30). While ethical considerations surrounding embryo selection remain, PGT represents an important preventive measure that clinicians should discuss with families facing the burden of XLCNM.

In addition to survival, quality of life remains a critical consideration in infants with XLCNM. Long-term ventilator dependence, feeding tube support, recurrent respiratory infections, and repeated hospitalizations impose significant physical and emotional burdens on both patients and families. Multidisciplinary management, including respiratory therapy, nutritional support, rehabilitation, and psychosocial counseling, may help optimize comfort and functional outcomes. Early genetic diagnosis also allows families to make informed decisions regarding long-term care planning and palliative strategies. These measures do not alter the underlying disease course but may improve supportive care and overall quality of life.

Taken together, our case and the literature review indicate the importance of integrating clinical evaluation, family history, molecular diagnostics, and supportive technologies to improve early detection and counseling. While curative therapies remain under investigation, timely genetic diagnosis can help families make informed decisions, guide reproductive planning, and ensure affected infants receive appropriate supportive and palliative care.

## Data Availability

The original contributions presented in the study are included in the article/Supplementary Material, further inquiries can be directed to the corresponding author.
